# Connected interactions: enriching food web research by spatial and social interactions

**DOI:** 10.1098/rstb.2023.0163

**Published:** 2024-07-22

**Authors:** Fernanda S. Valdovinos, Antonio Bodini, Ferenc Jordán

**Affiliations:** ^1^ Department of Environmental Science & Policy, University of California, Davis, CA, USA; ^2^ Department of Chemistry, Life Sciences and Environmental Sustainability, University of Parma, Parma, Italy

**Keywords:** food web, social network, landscape connectivity, socio-ecological systems, ecological networks

## Abstract

This theme issue features 18 papers exploring ecological interactions, encompassing metabolic, social, and spatial connections alongside traditional trophic networks. This integration enriches food web research, offering insights into ecological dynamics. By examining links across organisms, populations, and ecosystems, a hierarchical approach emerges, connecting horizontal effects within organizational levels vertically across biological organization levels. The inclusion of interactions involving humans is a key focus, highlighting the need for their integration into ecology given the complex interactions between human activities and ecological systems in the Anthropocene. The comprehensive exploration in this theme issue sheds light on the interconnectedness of ecological systems and the importance of considering diverse interactions in understanding ecosystem dynamics.

This article is part of the theme issue ‘Connected interactions: enriching food web research by spatial and social interactions’.

## 1. Introduction

The study of diverse and intricate networks of interactions among co-existing organisms lies at the heart of ecological research. Food webs have traditionally been the most studied of these networks [1,2], reflecting the fundamental ‘who eats whom’ in ecosystems [3,4]. Over time, food webs have been interpreted in various ways, from networks of matter and energy flows [5–9] to control flow networks where trophic relationships generate bottom-up and top-down forces [10–14]. In the last few decades, however, food web research has bloomed by embracing more complexity at all hierarchical levels, including different types of interspecific interactions, spatial and temporal scales and resolution of intraspecific interactions.

Much attention has turned recently to non-trophic interactions such as facilitation and mutualisms [15,16], which are now seen as pivotal to community organization and functioning [17–20], giving rise to the exciting new field of multiplex networks [20–24]. Furthermore, increased computing power, collaborations and data sharing, together with the rise of new technologies, is allowing ecologists to analyse the effect of spatial [25,26] and population structure [27–29] and physical variables [30,31] on the structure and dynamics of food webs. More recently, research opening new frontiers in network ecology is analysing how coupled natural-human systems would respond to environmental change, including in fisheries [30,32,33], ecosystem services [34,35] and human social conflicts [36].

The time is ripe now for studying connections between these diverse interactions (i.e. spatial, interspecific, intraspecific, anthropogenic and physical) affecting organisms integrating biological communities. Enriching community ecology by integrating spatial and social processes has traditionally been challenging owing to an epistemological bias towards horizontal thinking [37]. It is easier to describe and analyse relationships at the same organizational level, such as species linked by predation, individuals linked by dominance or habitat patches linked by dispersal. Methodologies and sampling techniques have been more advanced for these horizontal perspectives, evident in food webs, social networks and landscape networks. Additionally, research institutions and conferences have typically been organized by horizontal disciplines (e.g. biochemistry, ethology and ecology), making vertical integration culturally and structurally more difficult. However, there is now significant progress in vertical integration. For example, research in food webs [38,39] and mutualistic networks [40–44] has evaluated the effect of adaptive foraging occurring at the individual level on community-level variables such as network structure and stability and species persistence, as well as ecosystem-level variables such as secondary productivity and pollination services. This progress is driven by advancements in network science, computational simulations, mathematical and statistical models and interdisciplinary collaborations, which are beginning to bridge the gaps between traditional horizontal perspectives and the vertical integration of ecological processes. This theme issue showcases such progress by presenting papers that enhance our understanding of connections across organizational levels and spatial scales.

**Figure 1.**
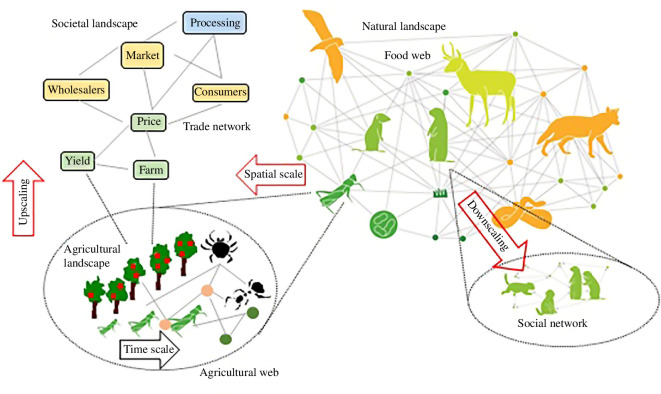
Connected connections within the hierarchy of individuals, social groups, species and their spatial and societal linkages. Interacting processes highlight the importance of downscaling and upscaling network composition.

To illustrate how these interactions are co-occurring and therefore potentially interconnected, we start our argument from an organism in a population (figure 1). It responds to other individuals within its population in a way that depends on the population features, such as age-structure and social network. This organism also interacts with organisms of other species, shaping a web of positive (mutualism or facilitation), negative (competition) and mixed (predation, parasitism) interactions. Spatial heterogeneity and cultural landscapes shape different communities so that organisms become actors in different webs that may change in time. Finally, in the Anthropocene, human activity alters the survival and reproduction of this focal organism, as well as the spatial and temporal structure of the abiotic and biotic processes affecting its intraspecific and interspecific interactions. This human activity is not just an external forcing at local scales but also interacts with the biotic and abiotic processes of the ecological system in which our focal organism lives.

In summary, trophic and non-trophic, spatial and intraspecific processes as well as interactions with human activities collectively shape the co-existence of diverse organisms and the dynamics of ecological systems. This theme issue focuses on progress connecting these complex interactions to provide a comprehensive understanding of ecological systems. In the rest of this introduction to the theme issue, we present the enclosed papers organized by five themes that have different aspects of vertical integration, which are (i) connecting metabolic interactions among unicellular organisms to global distributions in marine ecosystems; (ii) the effect of intraspecific social interactions on populations and communities; (iii) novel approaches to decipher the structure of ecological networks across space; (iv) connecting and/or comparing local networks across space; and (v) understanding the structure and dynamics of coupled human–natural systems.

## 2. Topics organizing this theme issue

### (a) From metabolic interactions among unicellular organisms to global distributions in marine ecosystems

Phytoplankton are fundamental to aquatic ecosystems, acting as primary producers that support oceanic food webs and drive biogeochemical cycles. Their photosynthetic processes contribute significantly to global primary production and carbon sequestration, playing a vital role in regulating the Earth’s climate [45–47]. The interactions and productivity of these microscopic organisms influence biological organization at all hierarchical levels, from individual cells to populations, communities and broader ecosystems. This theme issue includes studies [48–50] which explore the intricate dynamics of phytoplankton within marine ecosystems using advanced DNA-based techniques, global databases and innovative statistical methods.

Nef *et al*. [48] (P1) focus on the metabolic interactions within phytoplankton communities and their significance in global marine networks. By employing a range of methods from ecophysiology to omics data analysis, they unravel the complex interdependencies of phytoplankton species and their roles in nutrient cycling. This study sets the stage for understanding how these metabolic interactions influence broader ecological processes and community dynamics. In the second study on this theme, Bellardini *et al*. [49] (P2) broaden the scope by investigating the spatiotemporal changes in pelagic food webs using environmental DNA metabarcoding. This technique allows for the identification of community composition across different times and locations. By integrating eDNA data with connectivity analysis, this study reveals how ocean currents influence the distribution and movement of planktonic communities, affecting higher trophic levels. This work underscores the importance of combining eDNA with environmental factors and connectivity models to understand the seasonal dynamics and spatial homogeneity of marine ecosystems. Finally, Gaudin *et al*. [50] (P3) extend the scope to global distributions by examining the biogeographical signatures and ecological associations of marine plankton through DNA-based techniques. They integrate Association Distribution Modeling (ADM) with metagenomics data to identify major marine biomes, each with distinct community structures and sensitivities to environmental change. Their projections under climate scenarios suggest a reconfiguration of ecological associations and community connectivity, potentially altering the functional dynamics of these ecosystems, particularly in carbon fixation pathways. Gaudin *et al*. [50] advocate for integrating ADM with metabolic modelling to enhance our predictive capabilities regarding the evolution of marine ecosystems under climate change.

Beyond phytoplankton, Ito *et al*. [51] (P4) in this theme issue also integrate processes across different levels of organization but considering the entire food web in a mesocosm experiment. The authors assessed how sequential sublethal heatwaves affect a temperate benthic ecosystem, from individual physiological reactions to population biomass shifts and ecosystem-level carbon flux alterations of the dozen species composing the food web in the mescosm. They found that gastropods exhibited physiological stress memory, showing acclimation to repeated heatwaves, which increased their tolerance to future heat stress events. Moreover, the intensity of ecosystem carbon fluxes initially decreased after a single heatwave but increased after three sequential heatwaves, indicating acclimation at the ecosystem level. Changes in biomass and feeding preferences among trophic groups further illustrate the adaptive strategies that maintain resilience within the trophic network.

Together, these studies illustrate the power of advanced molecular tools, mesocosm experiments and integrative approaches in uncovering the complex dynamics of marine ecosystems. By making significant progress in vertical integration, they enhance our understanding of how different organizational levels interact across time and space.

### (b) The effect of intraspecific interactions on populations and communities

Interactions among organisms of the same population can influence how organisms of different species interact with each other in a community context. Three studies in this theme issue [52–54] focus on the influence of social interactions within a population on its interspecific interactions. The studies by Bronstein & Sridhar [52] and Madsen & de Silva [54] both review prior literature to build new conceptual frameworks. Specifically, Bronstein & Sridhar [52] (P5) develop a conceptual framework to understand how cooperation within social species affects their mutualistic interactions with other species. They suggest that collaborative behaviours within a species can enhance the efficiency and effectiveness of mutualistic partnerships. For example, eusocial insects like bees use the waggle dance to convey precise information about resource locations, benefittting their plant partners through effective pollination. Similarly, ants farm fungi or tend to aphids, activities requiring high levels of cooperation to ensure colony success and mutualistic benefits. This framework highlights how social structures and behaviours within a species can significantly alter outcomes of interspecific interactions, emphasizing a complex interdependence central to ecological and evolutionary patterns.

Ogino & Farine [53] (P6) develop an individual-based model to evaluate the effect of collective intelligence on resource partitioning. They use computational simulations to evaluate how collective intelligence influences resource partitioning in animal groups. Their study reveals that group living enhances foraging efficiency through collective decision making and frequency-dependent learning. Larger groups develop more distinct foraging preferences, especially in diverse environments, pointing to the role of collective intelligence in fostering foraging specialization and shaping group size and territorial behaviours. The origins of sociality might be closely linked to the interplay between individual and collective information processing and memory dynamics.

Madsen & de Silva [54] (P7) discuss fission–fusion dynamics using the framework of complex adaptive systems (CAS). Fission–fusion dynamics describe flexible social structures where group composition and size change in response to environmental and social factors. This perspective shifts the focus from static definitions to the processes driving social organization, accommodating the fluidity and variability in such societies. These dynamics can affect how species share or compete for resources, encounter predators or prey and use space. For instance, changes in group size and composition can influence resource use patterns, territorial behaviour and disease or information transmission between species.

Together, these studies illustrate how intraspecific cooperation, flexible social structures and collective intelligence can influence how species interact with each other and their environment. By examining these intraspecific interactions, the studies advance our understanding of vertical integration in ecological research, highlighting the interconnectedness of individual behaviours, population dynamics and community-level outcomes.

### (c) Novel approaches to decipher the structure of ecological networks across space

Our understanding of how ecological networks vary across spatial scales is currently limited by the complexity of acquiring repeated spatial data for species interactions. Moreover, the construction of species interactions networks is fraught with challenges such as sampling bias, which might overlook rare or cryptic species, and the dynamic nature of species interactions that can vary over time and space. Metawebs can address these limitations by aggregating all known interactions across various habitats and times, offering a more inclusive and extensive representation of potential trophic (and non-trophic) interactions [55]. They fill in gaps in data by including interactions involving rare or less observable species and can adapt to account for emerging interactions as habitats are altered by human activity. They also provide a larger dataset useful for testing ecological theories and models and offer valuable insights for conservation efforts by identifying key species and interactions that are crucial for maintaining ecosystem health and resilience. In this theme issue, Hale *et al*. [55] and Dansereau *et al*. [56] show how much ecology can gain from metawebs when creatively combining them with other global databases, statistical methods and theoretical models.

Hale *et al*. [55] (P9) built the most resolved aboveground terrestrial food web with approx. 580 000 feeding links among approx. 3800 taxonomic species using a wide range of data sources, including public records, occurrence data and expert knowledge. By comparing this metaweb to the expectations of the niche model, the study finds that its structure qualitatively deviates from previous food webs (most of them aquatic and a few coarsely resolved terrestrial) owing to the specific structural properties of terrestrial herbivores. This deviation suggests that terrestrial herbivory is structured by processes different from those in aquatic systems.

The metaweb approach to be useful across spatial scales needs to account for the variability of species interactions across different environmental conditions and geographical locations. Dansereau *et al*. [56] (P8) address this gap by developing a probabilistic framework for downscaling metawebs to more accurately reflect the ecological contexts of local communities. This innovative framework involves constructing a regional metaweb, integrating species distribution models (SDMs) to gauge the potential presence of species within various ecoregions, and then spatially refining the metaweb data in conjunction with these SDMs. This allows for the prediction of local community compositions from the broader metaweb and for estimating the likelihood of each interspecific interaction in the local community, which is an important advance for network ecology as our knowledge of many interspecific interaction is uncertain. This methodology was designed with an emphasis on future empirical validation, where actual food web data could be used to refine and improve the model’s predictive accuracy. This approach is promising for identifying areas where ecological networks exhibit unique characteristics or where conservation efforts should be focused. There are several systems, however, in which identifying all species and their interactions to build a metaweb is unfeasible for the time and sampling effort available to a research team. Kininmonth *et al*. [57] (P10) in this issue used spatiotemporal co-occurrence data captured by photography to infer species interactions for benthic communities of the Spanish coastal zone across eight sites, five depths and sunlit/shaded aspects, which would have been prohibitive through other methods. The authors employ Exponential Random Graph Models to infer the network of relationships from species’ statistical preferences for specific neighbours, based on the sequence in which organisms of different species in the photography (i.e. attachment preferences), influenced by and varying with spatial scales. This analysis is used to measure ‘co-occurrence social diversity’ of each site as the proportion of structural groups within such network of attachment preferences, which complements alpha diversity by providing information on preferential interactions between species not just species composition.

Together, these studies illustrate the power of novel methodologies and integrative approaches in uncovering the structure of ecological networks across spatial scales. By leveraging metawebs, probabilistic frameworks and innovative data collection techniques, they provide a deeper understanding of how species interactions and network configurations vary across different environments and geographical locations.

### (d) Connecting and/or comparing local networks across space

Understanding how local networks are spatially connected is crucial for elucidating how environmental changes impact species interactions and network configurations in heterogeneous landscapes. Moreover, comparing local networks across different levels of human intervention across space provides an important understanding on how human activity impacts ecological networks. This theme issue includes the studies by Jordán *et al.*, Henriksen *et al.* and Agnetta *et al.* [26,58,59], which explore the effects of environmental changes, habitat fragmentation and human activities on local ecological networks. These studies highlight the importance of examining networks across space to uncover the nuanced effects that might be masked when only studying local networks isolated from their spatial structure.

Jordán *et al*. [26] (P11) studied six subnetworks representing different regions of the Barents Sea to understand how atlantification affects these areas. They found that atlantification, characterized by the northward expansion of fish stocks like cod, haddock and redfish, led to increased modularity in the northern subnetworks and decreased modularity in the southern subnetworks over time. This indicates that the northern parts of the Barents Sea are experiencing more direct effects of atlantification, including changes in species composition and interactions within those food webs. The division into subnetworks provides a nuanced view of how environmental changes impact ecological networks at smaller scales.

Henriksen *et al*. [58] (P12) used a multi-layer network approach to determine species roles within plant-pollinator networks in a landscape of remnant semi-natural grasslands. Their approach identified specific plant and bee species that served as connectors among patches, playing a crucial role in the cohesion of fragmented habitats. They also evaluated network turnover across fragments using beta diversity of species and their interactions. They found that road verges had lower beta diversity than semi-natural grasslands and that beta diversity increased with the patch size of grassland fragments. Their study suggests that road verges may not be as effective in supporting habitat connectivity.

Agnetta *et al*. [59] (P13) evaluated the impact of fishing on fish trophic positions by comparing stable isotope analysis results between fishery-restricted areas and trawled areas. The analysis revealed that fish from heavily fished areas had lower trophic levels compared with those from fishery-restricted areas. This suggests that fishing activities can indirectly alter food web structure by impacting the trophic levels of fish, which may have broader ecological implications for marine ecosystems.

Together, these studies illustrate the importance of examining local ecological networks within their spatial context to understand the impacts of environmental changes and human activities. By embedding spatial structure into subnetworks, using multi-layer network approaches and comparing stable isotope analysis results across different levels of human intervention, they provide a comprehensive view of how species interactions and network configurations vary across heterogeneous landscapes.

### (e) Understanding the structure and dynamics of coupled human–natural systems

The complexity of socio-ecological systems makes it extremely challenging to understand the interconnected processes acting in parallel. These systems involve numerous drivers, such as overfishing and pollution, that result in various effects, including pollination crises and climate change. A key question in this framework is how the complex dynamics of multiple interactions determine the response of ecosystems to management and human interventions. This theme issue includes the studies by Kushal & Springborn, Bodini *et al.*, Ortiz & Hermosillo-Núñez and Biswas *et al.* [33,36,60,61], which explore the intricate relationships between human activities and natural systems, offering insights into sustainable management and policy design.

Kushal *et al*. [33] (P14) investigate the impact of different fishery policies by incorporating fishers with varying levels of success as additional nodes in networks representing marine food webs. Their study simulates the effects of these policies on target and bycatch species, addressing the dynamic interactions within the marine food web, including human harvest impacts. By merging ecological networks with bio-economic elements, they provide a quantitative assessment of fishery management policies, exemplifying the principles of Ecosystem-Based Fisheries Management.

Ortiz *et al*. [60] (P15) expand the concept of keystone complexes to include eco-social keystone complexes, where keystone properties are influenced by social factors. By identifying core eco-social components, their study facilitates the design and assessment of sustainable management strategies for marine ecosystems. This approach represents a significant advancement over traditional fisheries management strategies that focus solely on economically valuable species, highlighting the importance of considering social factors in ecological management.

Bodini *et al*. [36] (P16) use networks to map interactions among social and ecological variables influencing the long-standing armed conflict in Colombia. Applying loop analysis to these networks, they predict the system’s response to various policy interventions, revealing the complexity of achieving synergies and navigating trade-offs between ecological conservation and social development goals. This approach uncovers causal pathways, offering valuable insights into how policies might produce intended or unintended outcomes, emphasizing the need for careful consideration of complex ties in planning and policy-making to promote sustainable development within human–natural systems.

Biswas *et al*. [61] (P17) explore the spatial dimensions of multi-species trophic networks in urban ecosystems, focusing on how scavenger communities are organized in terms of interactions and sampling sites. Their study highlights the role of scavenging as a crucial mechanism in urban areas, showing how urban expansion affects behavioural adaptations at individual and population levels, as well as the organization of multi-species scavenger communities. Key players in this system include free-ranging dogs and the common myna bird, assigning significant roles to species typically considered as outliers.

Together, these studies illustrate the power of integrating social and ecological factors to understand the structure and dynamics of coupled human–natural systems. By employing innovative network approaches and considering the impacts of human activities, they provide comprehensive insights into sustainable management and policy design. This theme issue underscores the importance of examining the complex interactions within socio-ecological systems to effectively address environmental and social challenges, promoting resilience and sustainability.

## 3. Conclusions: what have we learned?

**Figure 2.**
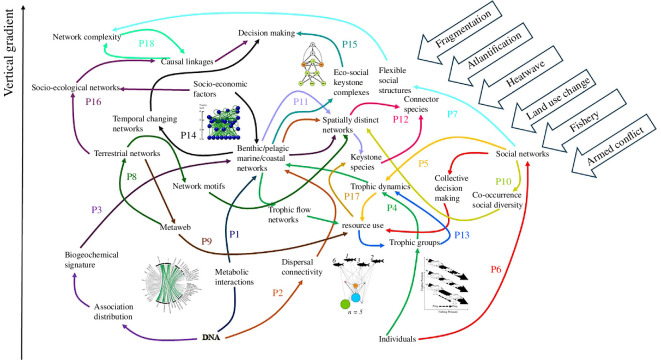
Connections of the 18 papers of this theme issue along a vertical integration gradient. Papers are labelled from P1 to P18 according to the text and colour lines depict the logical connectors between their subjects. On the top right, a few stress factors (drivers of change) are shown that are considered in the analyses.

Improving ecological research requires better integration and mechanistic coupling of various processes. This includes linking agents at similar organizational levels (‘horizontal’ effects) and connecting parts to wholes (‘vertical’ effects). Connecting individual behaviour, group dynamics, population structure, interspecific interactions, community dynamics and spatial processes is challenging but essential to develop a more comprehensive understanding of ecological systems and how they respond to environmental change. This theme issue presents studies that span from the metabolic interactions of unicellular organisms to the intricate dynamics of coupled human–natural systems, offering comprehensive insights into the complexity of ecological systems. The enclosed studies investigate the connections between interactions occurring at the cellular level all the way to global distributions, as well as the effect of intraspecific interactions (including social behaviours) on interspecific interactions. This theme issue also presents studies that investigate the differences of ecological networks across space in heterogeneous landscapes and the effect of connectivity on the local networks, as well as promising methods and interdisciplinary approaches that can promote horizontal and vertical connections for many types of ecological systems (figure 2).

Studies in this issue show that the complexity of socio-ecological systems necessitates an integrated approach to understand the interconnected processes driven by human activities and natural dynamics. By incorporating social factors, bio-economic elements and network approaches, we can address environmental and social challenges more effectively. Studies demonstrate how fishery policies and eco-social keystone complexes offer valuable insights for sustainable management and policy design, promoting resilience in human–natural systems.

In summary, this theme issue brings together 18 papers that collectively advance our understanding of ecological networks through the integration of diverse interactions, spatial scales and human influences. Together, these studies illustrate the transformative potential of vertical integration in ecological research. By bridging gaps between traditional horizontal perspectives and incorporating multiple organizational levels and spatial scales, we gain a more holistic understanding of ecological networks. This theme issue underscores the importance of interdisciplinary collaborations and innovative methodologies in advancing our knowledge of ecological systems, ultimately informing more effective conservation and management practices. However, as Damos [62] (P18) questions in this issue: are ecological networks reliable and unbiased logical representations of true causal flows within complex systems? Or are they simply products of researchers’ compromises with simplified models, statistical assumptions and intuitive knowledge? We must acknowledge that causal ecological networks are subject to initial rules and data characteristics and will never fully capture the intricate complexities of the systems they represent. Scholars must progress in understanding connections and their implications while recognizing limitations and assumptions to make the science of connections more comparative and reliable.

## Data Availability

This article has no additional data.
